# SIR Model for Dengue Disease with Effect of Dengue Vaccination

**DOI:** 10.1155/2018/9861572

**Published:** 2018-08-28

**Authors:** Pratchaya Chanprasopchai, I. Ming Tang, Puntani Pongsumpun

**Affiliations:** ^1^Department of Mathematics, Faculty of Science, King Mongkut's Institute of Technology Ladkrabang, Chalongkrung Road, Ladkrabang, Bangkok 10520, Thailand; ^2^Computational and Applied Science for Smart Innovation Cluster (CLASSIC), Faculty of Science, King Mongkut's University of Technology Thonburi, Bangkok 10140, Thailand

## Abstract

The dengue disease is caused by dengue virus, and there is no specific treatment. The medical care by experienced physicians and nurses will save life and will lower the mortality rate. A dengue vaccine to control the disease is available in Thailand since late 2016. A mathematical model would be an important way to analyze the effects of the vaccination on the transmission of the disease. We have formulated an SIR (susceptible-infected-recovered) model of the transmission of the disease which includes the effect of vaccination and used standard dynamical modelling methods to analyze the effects. The equilibrium states and their stabilities are investigated. The trajectories of the numerical solutions plotted into the 2D planes and 3D spaces are presented. The main contribution is determining the role of dengue vaccination in the model. From the analysis, we find that there is a significant reduction in the total hospitalization time needed to treat the illness.

## 1. Introduction

**Figure 1:**
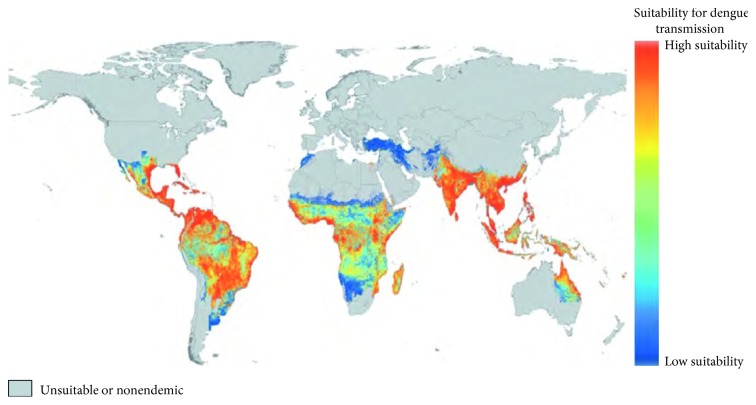
Distribution of global dengue risk [2].

Dengue disease is a mosquito-borne viral infection caused by 4 serotypes of dengue virus, DEN-1, DEN-2, DEN-3, and DEN-4. Dengue disease is widely spread in tropical and subtropical regions of the world. Dengue virus is transmitted to human by the bite of the female mosquito of the species *Aedes aegypti* and *Aedes albopictus* [1]. An estimated 3.9 billion people in 128 countries are at risk to this disease. The countries at danger to infection by the dengue viruses around the world are shown in Figure 1 [2].

Thailand is located in the tropical region where dengue virus is widely circulating. Dengue is spreading nationwide in Thailand including the Bangkok metropolitan area. Thailand is in special danger since three of the four species of the dengue virus have been found in Thailand, and both of the *Aedes* vector species are present. The Bureau of Epidemiology, Ministry of Public Health, has reported dengue cases in all provinces in 2016, a total of 63,931 cases with 64 deaths [3]. At the present time, there is no special treatment for dengue disease, but early detection and the appropriated medical care will decrease the fatality rates. A dengue vaccine would be another way to reduce the fatality rates. WHO reported the first dengue vaccine, called as Dengvaxia (CYD-TDV). It was registered in several countries in late 2015 and early 2016. It was recommended for use only in high dengue disease burden countries such as Thailand [1]. Dengue vaccine against four strains of the dengue virus was first launched in Thailand in late 2016. The vaccine would be suitable for use in individuals between 9 and 45 years of age living in endemic areas. Since the reported incidence of dengue peaks in the rainy season between June and September, the vaccination should be done in advance of the peak period in order for the immunity to develop.

There were many mathematical models for describing and analyzing the behaviors of dengue disease. Esteva and Vargus [4] proposed an SIR (susceptible-infected-recovery) model to describe the transmission of dengue disease with constant human and vector populations while Chanprasopchai et al. [5] proposed a SEIR (susceptible-exposed-infected-recovered) model for Thailand to determine the effect of the rainfall on the spread of dengue in the Thailand model. The transmission of dengue disease is assumed to depend on the nature of the rainfall in different countries. The stability of the solution of the model was then analyzed. Numerical results taking into account the rainfall were obtained, and they were seen to correspond to the analytical results. Using standard dynamical analysis techniques, Chanprasopchai and Pongsumpun [6] established relations between the different variables in an SIR model of the dengue transmission model in which the biting rate of mosquito became as factor. Pongsumpun and Tang [7] analyzed the transmission of dengue hemorrhagic fever in an SIR model which included an age structure in human population.

Recently, Shim [8] studied the recently approved dengue vaccination program in the Philippines and showed that with appropriated pricing of dengue vaccination, reduction of the burden of the dengue disease in the Philippines and a significant potential to confer the excellent value were possible. Recker et al. [9] reported that the availability of epidemiological and clinical data from the trials of vaccine provided a great opportunity for formulating mathematical models in which the vaccine efficacy depends on the serotype, age, host immune status, and severity. Mathematical modelling becomes a valuable tool in the policy-making process to estimate what the consequences of any decisions taken could be. Aguiar et at. [10] studied the impact of the tetravalent dengue vaccine (Dengvaxia™) in the dengue endemic countries. They divided the human population into four age groups (below 9 years, between 9 and 45 years and naturally infected with dengue fever, between 9 and 45 years and vaccinated, and older than 45 years). The reasons for dividing the humans into these four groups are the differences in the observed response of patients of different ages to the vaccine. Sridhar et al. [11] reported that the effect of the vaccine was different for people in these four groups. Aguiar et al. [10] also took into account the presence of two strains of the dengue virus in their model even though the vaccine was made with attenuated viruses of the four serotypes of the dengue virus (DEN-1, DEN-2, DEN-3, and DEN-4). In their model, there were thirty-one (31) population categories with differential equations defining the time evolution of each category. In their model, however, the transmission of the dengue virus was taken to be due to a contact between a susceptible person and an infected person even though dengue fever is abovirus disease where the transmission occurs when a susceptible vector (mosquito) bites an infected human and then inturn the infected mosquito bites a susceptible human. Sriprom et al. [12] have studied the transmission of DF when there are two dengue viruses present and no age structure in the human population and where the transmission of disease is via the mosquitoes. The transmission of a single strain of the virus in an age-structured human population has been studied by Pongsumpun and Tang [7].

In this study, we propose an SIR mathematical model to analyze the behaviors of the transmission of dengue fever when vaccination with the Dengvaxia has been carried out. To be able to conduct a simple analytical analysis of the results, only one strain of the DF virus was taken into account and no age structure in the human populations was assumed. Otherwise the mathematics would become intractable. Only inferences based on the numerical simulations of the solutions to the multiple differential equations (31 for the model used in [10]) describing the time evolution of the many population categories can be drawn. The impact of the vaccination on the transmission in our model is expressed through the parameter *α* (the efficacy) appearing in (1). We note that this parameter appears in most of the analytical expressions for the various population categories and the basic reproduction rate *R*_0_. The standard analysis method using the Routh–Hurwitz criteria is applied to investigate the system stability in which the dynamical transmission model of dengue disease, equilibrium state, stability, numerical simulation, results, and conclusion are presented.

## 2. Materials and Methods

**Figure 2:**
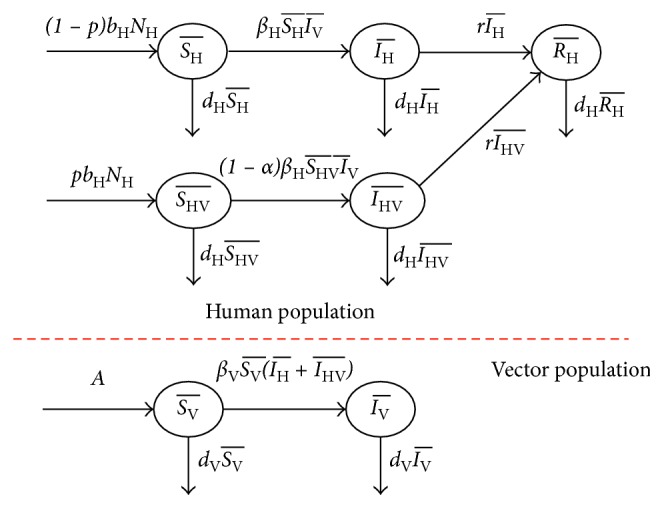
Dynamical transmission in humans and mosquitoes with the effect of vaccination incorporated. *Note.*$\bar{S_{\text{H}}}\left(t\right)$ = number of susceptible human population who are unvaccinated at time *t*; $\bar{I_{\text{H}}}\left(t\right)$ = number of infected human population who are unvaccinated at time *t*; $\bar{R_{\text{H}}}\left(t\right)$ = number of recovered human population who are unvaccinated at time *t*; $\bar{S_{\text{HV}}}\left(t\right)$ = number of susceptible human population who have been vaccinated at time *t*; $\bar{I_{\text{HV}}}\left(t\right)$ = number of infected human population who have been vaccinated at time *t*; $\bar{S_{\text{V}}}\left(t\right)$ = number of susceptible vector at any time *t*; $\bar{I_{\text{V}}}\left(t\right)$ = number of infected vector at any time *t*; *d*_H_, *d*_V_ = death rates of human and vector populations; *N*_H_, *N*_V_ = total human and vector populations; *β*_H_, *β*_V_ = transmission rate of dengue virus from vector to human and human to vector; *b*_H_ = birth rate of human population; *A* = constant recruitment rate of vector population; *α* = vaccine efficacy; *p* = fraction of newborns vaccinated.

In our SIR model, the population is divided into 2 populations, a human and a vector population. The human population consists of three epidemiological states: susceptible humans ($\bar{S_{\text{H}}}$), infected humans ($\bar{I_{\text{H}}}$), and recovered humans ($\bar{R_{\text{H}}}$), while the vector population has two epidemiological states: susceptible vector ($\bar{S_{\text{V}}}$) and infected vector ($\bar{I_{\text{V}}}$). Mosquito has no recovery state since the mosquito dies before it can recover from the disease. The susceptible mosquito state is unimmune and uninfected, while in the infected state, it is infected with dengue virus and can transmit the virus. The recovery state in the human population is a person who has recovered from an infection by the dengue virus. We assume that the human and vector populations are constant. The dynamical transmission in human and mosquito population with effect of vaccination is shown in Figure 2.

The transmission model of dengue disease with effect of vaccination can be described by the following differential equations:

(1) $$\begin{array}{c} \frac{d\bar{S_{\text{H}}}}{dt}=\left(1-p\right)b_{\text{H}}N_{\text{H}}-\beta_{\text{H}}\bar{S_{\text{H}}}\bar{I_{\text{V}}}-d_{\text{H}}\bar{S_{\text{H}}},\\ \frac{d\bar{I_{\text{H}}}}{dt}=\beta_{\text{H}}\bar{S_{\text{H}}}\bar{I_{\text{V}}}-\gamma \bar{I_{\text{H}}}-d_{\text{H}}\bar{I_{\text{H}}},\\ \frac{d\bar{R_{\text{H}}}}{dt}=\gamma \left(\bar{I_{\text{H}}}+\bar{I_{\text{HV}}}\right)-d_{\text{H}}\bar{R_{\text{H}}},\\ \frac{d\bar{S_{\text{HV}}}}{dt}=pb_{\text{H}}N_{\text{H}}-\left(1-\alpha \right)\beta_{\text{H}}\bar{S_{\text{HV}}}\bar{I_{\text{V}}}-d_{\text{H}}\bar{S_{\text{HV}}},\\ \frac{d\bar{I_{\text{HV}}}}{dt}=\left(1-\alpha \right)\beta_{\text{HV}}\bar{S_{\text{H}}}\bar{I_{\text{V}}}-\gamma \bar{I_{\text{HV}}}-d_{\text{H}}\bar{I_{\text{HV}}},\\ \frac{d\bar{S_{\text{V}}}}{dt}=A-\beta_{\text{V}}\bar{S_{\text{V}}}\left(\bar{I_{\text{H}}}+\bar{I_{\text{HV}}}\right)-d_{\text{V}}\bar{S_{\text{V}}},\\ \frac{d\bar{I_{\text{V}}}}{dt}=\beta_{\text{V}}\bar{S_{\text{V}}}\left(\bar{I_{\text{H}}}+\bar{I_{\text{HV}}}\right)-d_{\text{V}}\bar{I_{\text{V}}}. \end{array}$$

The effect of the vaccination is incorporated into our model by the presence of the efficacy coefficient *α*. Setting it to 1, there will be no infected mosquitoes to transmit the virus and so there will be no dengue fever epidemic. This is however not the way the vaccination works. When a person is vaccinated with a vaccine made from live-attenuated virus, weak attenuated viruses are introduced into the human where the body immune system produces the agents which will provide immunity to further infection of the virus. Invariably, some people will not have an immune response able to the kill the weak virus, and weak virus will develop into a strong virus able to infect the person, producing an infectious person. The effect of the vaccination is to create a new pathway for the transmission of the disease.

The total human and vector populations are assumed to be governed by the following conditions:

(2) $$\begin{array}{c} \bar{S_{\text{H}}}+\bar{I_{\text{H}}}+\bar{R_{\text{H}}}+\bar{S_{\text{HV}}}+\bar{I_{\text{HV}}}=N_{\text{H}},\\ \bar{S_{\text{V}}}+\bar{I_{\text{V}}}=N_{\text{V}}. \end{array}$$

If total human and vector populations are constants, then the rate of change for total human and vector populations is 0. As the result, we will have the following equations:

(3) $$\begin{array}{c} \frac{d\bar{S_{\text{H}}}}{dt}+\frac{d\bar{I_{\text{H}}}}{dt}+\frac{d\bar{R_{\text{H}}}}{dt}+\frac{d\bar{S_{\text{HV}}}}{dt}+\frac{d\bar{I_{\text{HV}}}}{dt}=0,\\ \frac{d\bar{S_{\text{V}}}}{dt}+\frac{d\bar{I_{\text{V}}}}{dt}=0,\\ N_{\text{V}}=\frac{A}{\mu_{\text{V}}},\\ b_{\text{H}}=d_{\text{H}}. \end{array}$$

Normalizing the equations by introducing the following normalized variables:

(4) $$\begin{array}{c} S_{\text{H}}=\frac{\bar{S_{\text{H}}}}{N_{\text{H}}},\\ I_{\text{H}}=\frac{\bar{I_{\text{H}}}}{N_{\text{H}}},\\ R_{\text{H}}=\frac{\bar{R_{\text{H}}}}{N_{\text{H}}},\\ S_{\text{HV}}=\frac{\bar{S_{\text{HV}}}}{N_{\text{H}}},\\ I_{\text{HV}}=\frac{\bar{I_{\text{HV}}}}{N_{\text{H}}},\\ S_{\text{V}}=\frac{\bar{S_{\text{V}}}}{N_{\text{V}}},\\ I_{\text{V}}=\frac{\bar{I_{\text{V}}}}{N_{\text{V}}}. \end{array}$$

Introducing these normalized variables into (1), we get the new set of equations of the following states:

(5) $$\begin{array}{c} \frac{dS_{\text{H}}}{dt}=\left(1-p\right)b_{\text{H}}-\beta_{\text{H}}S_{\text{H}}I_{\text{V}}N_{\text{V}}-d_{\text{H}}S_{\text{H}},\\ \frac{dI_{\text{H}}}{dt}=\beta_{\text{H}}S_{\text{H}}I_{\text{V}}N_{\text{V}}-\gamma I_{\text{H}}-d_{\text{H}}I_{\text{H}},\\ \frac{dS_{\text{H}}}{dt}=pb_{\text{H}}-\left(1-\alpha \right)\beta_{\text{H}}S_{\text{HV}}I_{\text{V}}N_{\text{V}}-d_{\text{H}}S_{\text{HV}},\\ \frac{dI_{\text{HV}}}{dt}=\left(1-\alpha \right)\beta_{\text{H}}S_{\text{HV}}I_{\text{V}}N_{\text{V}}-\gamma I_{\text{HV}}-d_{\text{H}}I_{\text{HV}},\\ \frac{dI_{\text{V}}}{dt}=\beta_{\text{V}}S_{\text{V}}\left(I_{\text{H}}+I_{\text{HV}}\right)N_{\text{H}}-d_{\text{V}}I_{\text{V}}. \end{array}$$

The equilibrium states are obtained by setting the right-hand side of (5) to be 0. By doing this, we obtain an expression for something known as the basic production number *R*_0_. This number is defined as

(6) $$\begin{array}{c} R_{0}=\frac{\varepsilon_{1}\left(-\left(-2+\alpha \right)\varepsilon_{2}d_{\text{V}}+N_{\text{H}}\left(\left(1-p\alpha \right)d_{\text{H}}+\varepsilon_{3}N_{\text{V}}\beta_{\text{H}}\right)\beta_{\text{V}}\right)}{\sqrt{\varepsilon_{1}^{2}\left(\alpha^{2}\varepsilon_{2}^{2}d_{\text{V}}^{2}+2\alpha \varepsilon_{2}d_{\text{V}}N_{\text{H}}\left(\left(1+p\left(-2+\alpha \right)\right)d_{\text{H}}+\left(-1+2p\right)\varepsilon_{3}N_{\text{V}}\beta_{\text{H}}\right)\beta_{\text{V}}+N_{\text{H}}^{2}{\left(\left(-1+p\alpha \right)d_{\text{H}}+\varepsilon_{3}N_{\text{V}}\beta_{\text{H}}\right)}^{2}\beta_{\text{V}}^{2}\right)}}. \end{array}$$

When *R*_0_ ≤ 1, the equilibrium state will be the disease-free state *E*_1_ defined as

(7) $$\begin{array}{c} E_{1}\left(t\right)=\left(S_{\text{H}}=1-p,\;I_{\text{H}}=0,\;S_{\text{HV}}=p,\;I_{\text{HV}}=0,\;I_{\text{V}}=0\right), \end{array}$$

and when *R*_0_ > 1, the equilibrium state is the endemic state defined as

(8) $$\begin{array}{c} E_{2}\left(t\right)=\left(S_{\text{H}}^{*}\left(t\right),\;I_{\text{H}}^{*}\left(t\right),\;S_{\text{HV}}^{*}\left(t\right),\;I_{\text{HV}}^{*}\left(t\right),\;I_{\text{V}}^{*}\left(t\right)\right), \end{array}$$

where

(9) $$\begin{array}{c} S_{\text{H}}^{*}\left(t\right)=\frac{\varepsilon_{1}\left(\alpha \varepsilon_{2}d_{\text{V}}+\varepsilon_{3}\varepsilon_{4}\right)+\varepsilon_{5}}{2\alpha d_{\text{H}}\varepsilon_{6}},\\ I_{\text{H}}^{*}\left(t\right)=\frac{\varepsilon_{1}\left(-\alpha \varepsilon_{2}d_{\text{V}}+\left(1+\alpha -2p\alpha \right)\varepsilon_{4}\right)-\varepsilon_{5}}{2\alpha \varepsilon_{2}\varepsilon_{6}},\\ S_{\text{HV}}^{*}\left(t\right)=\frac{\varepsilon_{1}\left(-\alpha \varepsilon_{2}d_{\text{V}}+\varepsilon_{3}\varepsilon_{4}\right)+\varepsilon_{5}}{2\varepsilon_{3}\alpha d_{\text{H}}\varepsilon_{6}},\\ I_{\text{HV}}^{*}\left(t\right)=\frac{\varepsilon_{1}\left(\alpha \varepsilon_{2}d_{\text{V}}+\varepsilon_{3}\left(-1+2p\alpha \right)\varepsilon_{4}\right)-\varepsilon_{5}}{2\varepsilon_{3}\alpha \varepsilon_{2}\varepsilon_{6}},\\ I_{\text{V}}^{*}\left(t\right)=\frac{\varepsilon_{1}\left(-\left(-2+\alpha \right)\varepsilon_{2}d_{\text{V}}+\varepsilon_{3}\varepsilon_{4}\right)-\varepsilon_{5}}{2\varepsilon_{3}\varepsilon_{2}d_{\text{V}}N_{\text{V}}^{2}\beta_{\text{H}}^{2}}, \end{array}$$

with

(10) $$\begin{array}{c} \varepsilon_{1}=d_{\text{H}}N_{\text{V}}\beta_{\text{H}},\\ \varepsilon_{2}=\left(\gamma +d_{\text{H}}\right),\\ \varepsilon_{3}=\left(-1+\alpha \right),\\ \varepsilon_{4}=N_{\text{H}}N_{\text{V}}S_{\text{V}}\beta_{\text{H}}\beta_{\text{V}},\\ \varepsilon_{5}=\sqrt{d_{\text{H}}^{2}N_{\text{V}}^{2}\beta_{\text{H}}^{2}\left(\alpha^{2}{\left(\gamma +d_{\text{H}}\right)}^{2}d_{\text{V}}^{2}\right)}+\sqrt{2\left(-1+2p\right)\left(-1+\alpha \right)\alpha \left(\gamma +d_{\text{H}}\right)d_{\text{V}}N_{\text{H}}N_{\text{V}}S_{\text{V}}\beta_{\text{H}}\beta_{\text{V}}}+\sqrt{{\left(-1+\alpha \right)}^{2}N_{\text{H}}^{2}N_{\text{V}}^{2}S_{\text{V}}^{2}\beta_{\text{H}}^{2}\beta_{\text{V}}^{2}},\\ \varepsilon_{6}=N_{\text{H}}N_{\text{V}}^{2}S_{\text{V}}\beta_{\text{H}}^{2}\beta_{\text{V}}. \end{array}$$

The equilibrium states are local asymptotically stable if all the eigenvalues have negative real parts. The eigenvalues (*λ*) are obtained by solving the eigenvalue matrix equation

(11) $$\begin{array}{c} \det\left|J-\lambda I\right|=0, \end{array}$$

where *J* is the Jacobian matrix of each equilibrium point, *λ* is the eigenvalue, and *I* is the identity matrix.

The Jacobian matrix of system (6) is as follows:

(12) $$\begin{array}{c} J=\left[\begin{array}{ccccc} -\beta_{\text{H}}I_{\text{V}}N_{\text{V}}-d_{\text{H}} & 0 & 0 & 0 & -\beta_{\text{H}}S_{\text{H}}N_{\text{V}}\\ \beta_{\text{H}}I_{\text{V}}N_{\text{V}} & -\gamma -d_{\text{H}} & 0 & 0 & \beta_{\text{H}}S_{\text{H}}N_{\text{V}}\\ 0 & 0 & -\left(1-\alpha \right)\beta_{\text{H}}I_{\text{V}}N_{\text{V}}-d_{\text{H}} & 0 & -\left(1-\alpha \right)\beta_{\text{H}}S_{\text{HV}}N_{\text{V}}\\ 0 & 0 & \left(1-\alpha \right)\beta_{\text{H}}I_{\text{V}}N_{\text{V}} & -\gamma -d_{\text{H}} & \left(1-\alpha \right)\beta_{\text{H}}S_{\text{HV}}N_{\text{V}}\\ 0 & \beta_{\text{V}}S_{\text{V}} & 0 & \beta_{\text{V}}S_{\text{V}} & -d_{\text{V}} \end{array}\right]. \end{array}$$

Constructing the Jacobian matrix from (5) and evaluating it at the two equilibrium points, we obtain the eigenvalue equation

(13) $$\begin{array}{c} \left(-\lambda -\gamma -d_{\text{H}}\right)\left(\lambda^{4}+e_{1}\lambda^{3}+e_{2}\lambda^{2}+e_{3}\lambda^{1}+e_{4}\right)=0, \end{array}$$

for the disease-free state *E*_1_ and the eigenvalue equation

(14) $$\begin{array}{c} \left(\lambda^{5}+e_{1}\lambda^{4}+e_{2}\lambda^{3}+e_{3}\lambda^{2}+e_{4}\lambda^{1}+e_{5}\right)=0, \end{array}$$

for the endemic state *E*_2_.

The eigenvalues of disease-free equilibrium state will have negative real parts when the coefficients of (13) have values satisfying the Routh–Hurwitz criteria

(15) $$\begin{array}{c} e_{1}>0,\\ e_{3}>0,\\ e_{4}>0,\\ e_{1}e_{2}e_{3}>e_{3}^{2}+e_{1}^{2}e_{4}. \end{array}$$

The eigenvalues of the endemic equilibrium state will have negative real parts when the coefficients of (14) have values which satisfy a different Routh–Hurwitz criterion

(16) $$\begin{array}{c} e_{1}>0,\\ e_{2}>0,\\ e_{3}>0,\\ e_{4}>0,\\ e_{5}>0,\\ e_{1}e_{2}e_{3}-e_{3}^{2}-e_{1}^{2}e_{4}>0,\\ \left(e_{1}e_{4}-e_{5}\right)\left(e_{1}e_{2}e_{3}-e_{3}^{2}-e_{1}^{2}e_{4}\right)-e_{5}{\left(e_{1}e_{2}-e_{3}\right)}^{2}-e_{1}e_{5}^{2}>0. \end{array}$$

## 3. Numerical Results

**Figure 3:**
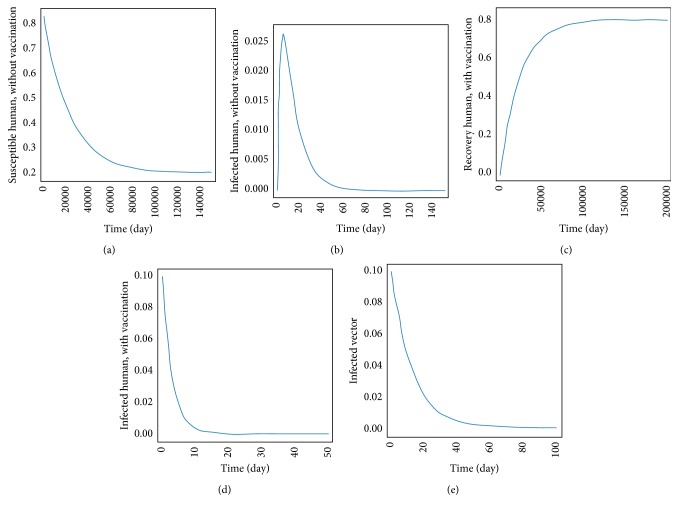
The trajectory of (a) *S*_H_, (b) *I*_H_, (c) *S*_HV_, (d) *I*_HV_, and (e) *I*_V_ towards the disease-free equilibrium state (*E*_1_).

**Figure 4:**
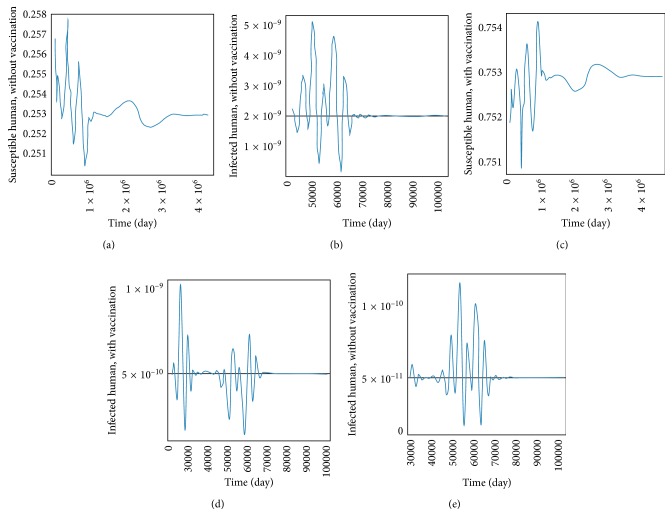
The trajectory of (a) *S*_H_, (b) *I*_H_, (c) *S*_HV_, (d) *I*_HV_, and (e) *I*_V_ towards the endemic equilibrium state (*E*_2_).

**Figure 5:**
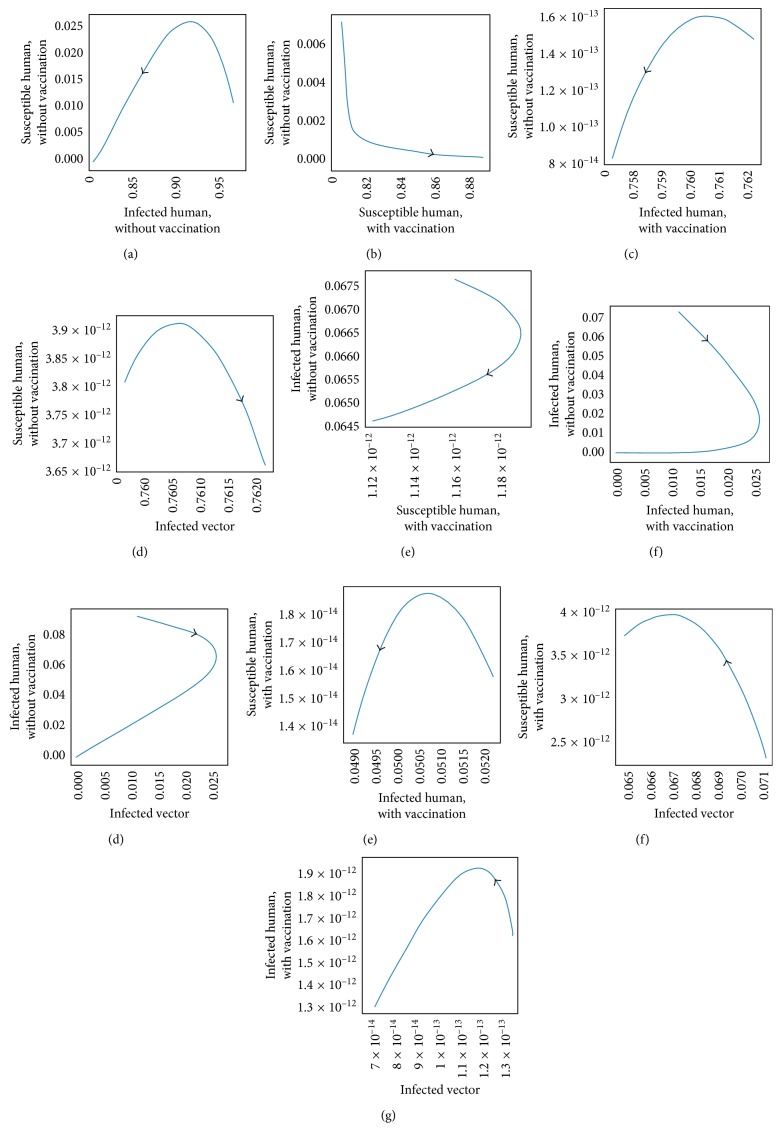
The trajectories of dengue disease for disease-free equilibrium projected onto (a) (*S*_H_, *I*_H_), (b) (*S*_H_, *S*_HV_), (c) (*S*_H_, *I*_HV_), (d) (*S*_H_, *I*_V_), (e) (*I*_H_, *S*_HV_), (f) (*I*_H_, *I*_HV_), (g) (*I*_H_, *I*_V_), (h) (*S*_HV_, *I*_HV_), (i) (*S*_HV_, *I*_V_), and (j) (*I*_HV_, *I*_V_) planes.

**Figure 6:**
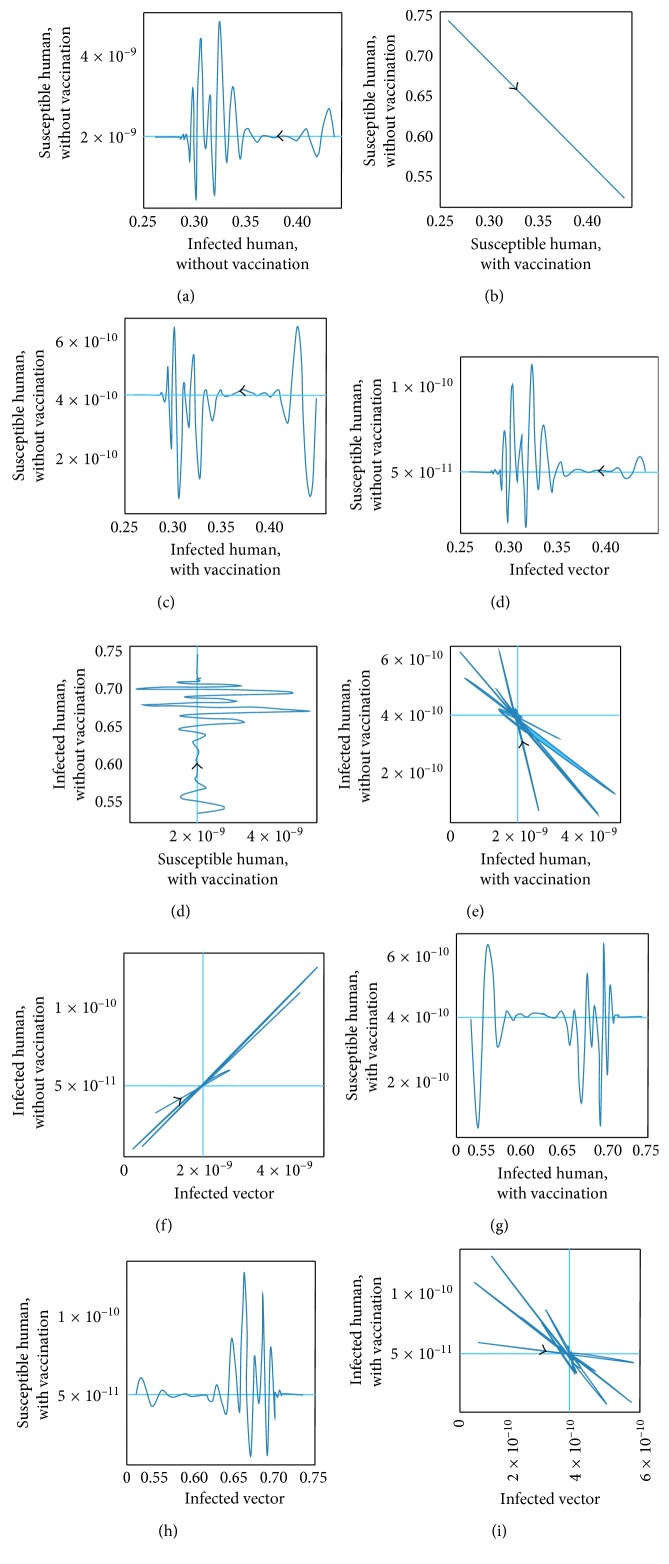
The trajectories of dengue disease for endemic equilibrium projected onto (a) (*S*_H_, *I*_H_), (b) (*S*_H_, *S*_HV_), (c) (*S*_H_, *I*_HV_), (d) (*S*_H_, *I*_V_), (e) (*I*_H_, *S*_HV_), (f) (*I*_H_, *I*_HV_), (g) (*I*_H_, *I*_V_), (h) (*S*_HV_, *I*_HV_), (i) (*S*_HV_, *I*_V_), and (j) (*I*_HV_, *I*_V_) planes.

**Figure 7:**
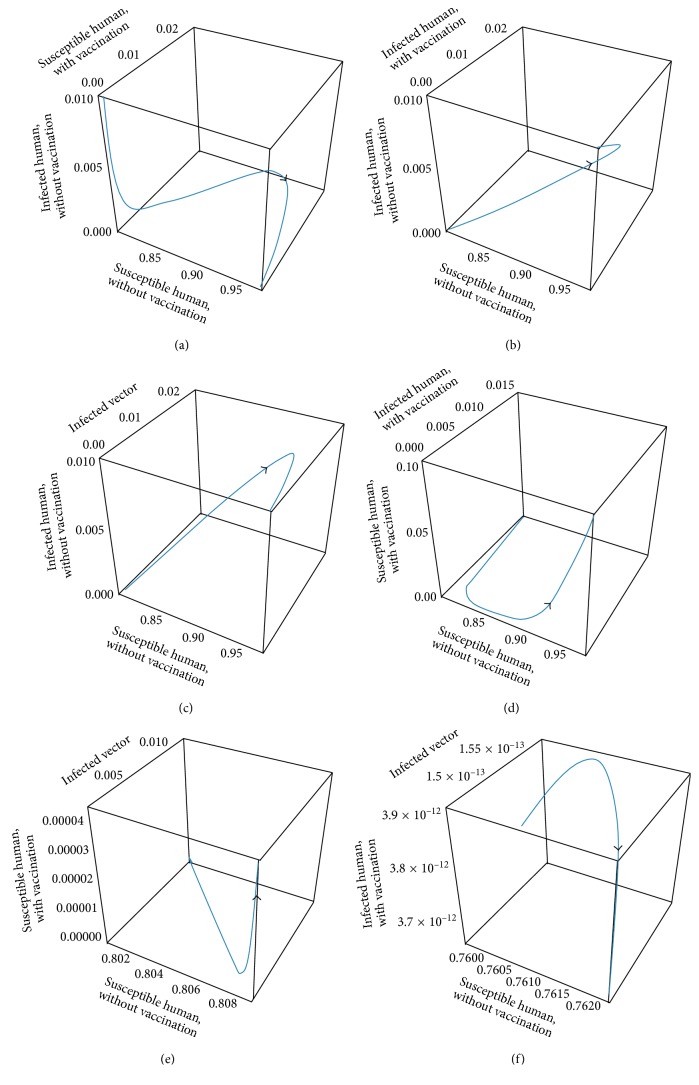
The trajectories of dengue disease for disease-free equilibrium projected onto (a) (*S*_H_, *I*_H_, *S*_HV_), (b) (*S*_H_, *I*_H_, *I*_HV_), (c) (*S*_H_, *I*_H_, *I*_V_), (d) (*S*_H_, *S*_HV_, *I*_HV_), (e) (*S*_H_, *S*_HV_, *I*_V_), and (f) (*S*_H_, *I*_HV_, *I*_V_) spaces.

**Figure 8:**
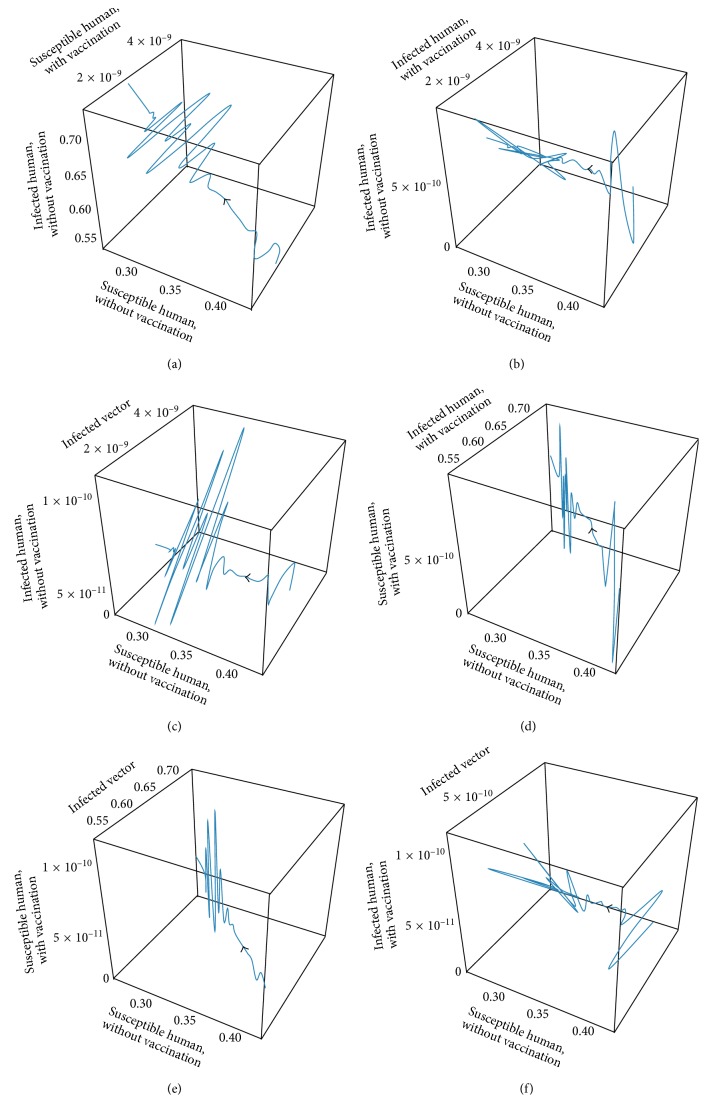
The trajectories of dengue disease for endemic equilibrium projected onto (a) (*S*_H_, *I*_H_, *S*_HV_), (b) (*S*_H_, *I*_H_, *I*_HV_), (c) (*S*_H_, *I*_H_, *I*_V_), (d) (*S*_H_, *S*_HV_, *I*_HV_), (e) (*S*_H_, *S*_HV_, *I*_V_), and (f) (*S*_H_, *I*_HV_, *I*_V_) spaces.

The transmission of dengue disease in this study is based on the SIR model with vaccination. The nonzero values of *α* and *p* are the parameters pertaining to the vaccination program. The numerical simulations were done using the following values of parameters: *d*_H_ = 1/(65^*∗*^365) per day corresponding to a life expectancy of 65 years for the Thai people and *d*_V_ = 1/12 corresponding to a life expectancy of 12 days of mosquito population. For the disease-free equilibrium state, the parameter values were *A* = 1,000; *N*_H_ = 1,000; *γ*_H_ = 1/3; *β*_H_ = 0.000012; *β*_V_ = 0.000012; *p* = 0.8; and *α* = 0.8, while the parameters value of the endemic equilibrium state were *A* = 500; *N*_H_ = 500; *γ*_H_ = 0.03; *β*_H_ = 0.000045; *β*_V_ = 0.000045; *p* = 0.75; and *α* = 0.75. These numerical values in the first set gave *R*_0_ < 1, while the values in the second set gave *R*_0_ > 1. The trajectories of the numerical simulations for disease-free and endemic states of *S*_H_, *I*_H_, *S*_HV_, *I*_HV_, and *I*_V_ are shown in Figures 3 and 4, respectively. The trajectories of the numerical simulation for disease-free and endemic states plotted in the 2D planes (*S*_H_, *I*_H_), (*S*_H_, *S*_HV_), (*S*_H_, *I*_HV_), (*S*_H_, *I*_V_), (*I*_H_, *S*_HV_), (*I*_H_, *I*_HV_), (*I*_H_, *I*_V_), (*S*_HV_, *I*_HV_), (*S*_HV_, *I*_V_), and (*I*_HV_, *I*_V_) planes are shown in Figures 5 and 6, respectively. The trajectories of the numerical solutions for disease-free and endemic states plotted in the 3D spaces (*S*_H_, *I*_H_, *S*_HV_), (*S*_H_, *I*_H_, *I*_HV_), (*S*_H_, *I*_H_, *I*_V_), (*S*_H_, *S*_HV_, *I*_HV_), (*S*_H_, *S*_HV_, *I*_V_), and (*S*_H_, *I*_HV_, *I*_V_) spaces are shown in Figures 7 and 8, respectively.

## 4. Discussion and Conclusion

In this study, the dynamical transmission of dengue disease based on an SIR model where a dengue vaccination campaign in the human population has occurred is studied. Again, it is found that the model system has two equilibrium points, a disease-free and an endemic state. The occurrence of the two equilibrium states depend on whether *R*_0_ < 1 and *R*_0_ > 1 where *R*_0_ is the basic reproduction number or number of secondary infection caused by an initial infection. The conditions for the stability of the disease-free and endemic equilibrium states were established.

It should be noted that the population was not divided into age groups or the presences of a second, third, or fourth serotype of the dengue virus were taken into account. The different population categories include all peoples between the age of one and sixty-five. The time series solutions of the disease-free and endemic equilibrium states are presented in Figures 3 and 4, respectively. In Figure 3, we see that the different population groups except for the recovered population decay monotonically to their equilibrium for *R*_0_ < 1.  Figure 4 shows that the time evolutions of the population categories oscillate before reaching their equilibrium values when *R*_0_ > 1. Interestingly, the solutions for infected populations with and without vaccination exhibit oscillations in two time intervals with the oscillation between two intervals becoming weak. The oscillation of the infected population without vaccination in first interval is stronger than that in the second time interval. This is opposite to the behavior of the oscillation of the infected population with vaccination. The trajectories of the disease-free and endemic equilibrium states onto 2D planes are shown in Figures 5 (when *R*_0_ < 1) and 6 (when *R*_0_ > 1), while the trajectories of the disease-free and endemic equilibrium states projected onto 3D planes are shown in Figures 7 (when *R*_0_ < 1) and 8 (when *R*_0_ > 1), respectively. In both the 2D and 3D projections, the trajectories towards the equilibrium values are smooth when *R*_0_ < 1. However, when *R*_0_ > 1, the trajectories exhibit oscillatory behavior in both the 2D and 3D projections. The conditions for the stability of disease-free and endemic equilibrium states were established. The time series solution of disease-free and endemic equilibrium states are presented in Figures 3 and 4, respectively. The trajectories of disease-free and endemic equilibrium projected onto 2D planes are shown in Figures 5 and 6, while the trajectories of disease-free and endemic equilibrium projected onto 3D planes are shown in Figures 7 and 8, respectively.

**Figure 9:**
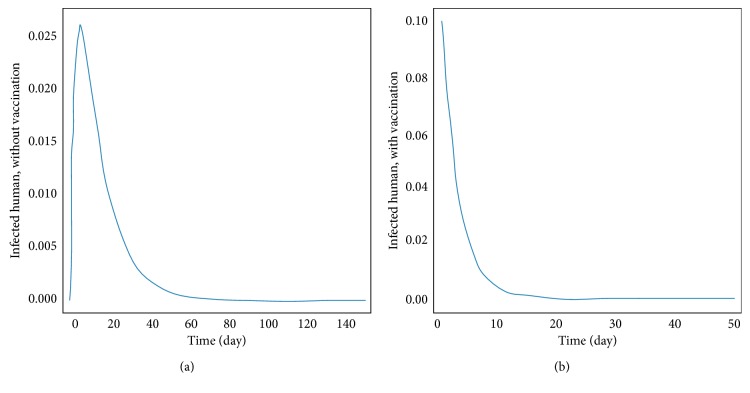
The infected human without vaccination (a) and with vaccination (b) by comparison of time series to disease-free equilibrium point.

**Figure 10:**
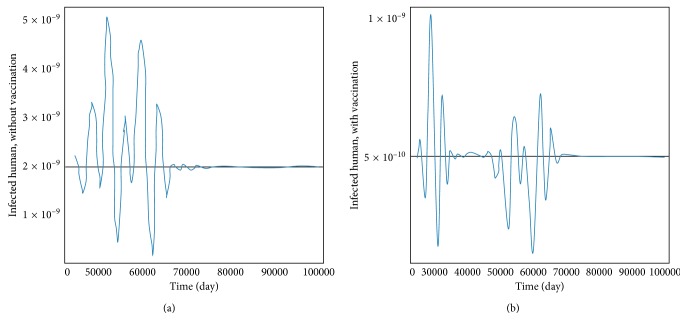
Infected human without vaccination (a) and with vaccination (b) by comparison of time series to endemic equilibrium point.

In order to analyze the effect of dengue vaccination, we have investigated both the disease-free and endemic equilibrium states using different values of parameters which would give *R*_0_ < 1 and *R*_0_ > 1. These same set of numerical values were used for numerical simulation with and without the influence of dengue vaccination campaign. *α* = 0 and *p* = 0 were used in the simulation to get the trajectories in the case where there was no vaccine administered. The influence of dengue vaccination is seen in Figures 9 and 10.  Figure 9 shows that the disease-free state is sooner when there are dengue virus vaccines administered than when there are not vaccines administered. This means that the hospitalization time can be reduced.  Figure 10 shows the effects of the vaccine when the parameters are such that the endemic state is in the equilibrium state.

The presence of oscillations around the endemic equilibrium state *E*_2_ means that the imaginary part of the eigenvalue is not zero. For the simulation shown in Figure 4, the imaginary part of the complex roots is approximately 0.000238428. This leads to an estimate of the period of the oscillations or *T*_period_ = 2*π*/*ω*, where *ω* = imaginary part of *λ* or 2*π*/0.000238428 ≈ 72.20 years. This value is the approximation to the period of the solutions [4].

In any vaccination campaigns, one must take into account the difference in the efficacy of the vaccine. It may not be the same for all age groups. Since one is not sure about the safety of the vaccine to children, the vaccination has been recommended only for people between the ages of 9 and 45. As of now, the vaccination schedule consists of 3 injections of 0.5 mL administered at 6-month intervals, given on a 0/6/12 month schedule [13].

The campaign in Thailand began in December 2016, and information on efficacy of the vaccine against the different serotypes and the difference in the efficacy for different age groups is being collected. Dengue disease in Thailand occurs in urban and suburban areas [14–17] with peak transmission rates during the rainy season [5, 18]. Seasonal and climate affect the dengue fluctuation [19–22]. At present, it is not recommended to give dengue vaccination to pregnant women and travelers or health-care workers at this time due to lack of sufficient data.

Since the present model does not take into account the age structure of the human population, the presence of more than one serotype of the dengue virus, and a proper treatment of how the vaccine interacts with a susceptible human being after it is administered to a human, one cannot answer the present question now facing the Public Health Community: Is the tetravalent vaccine safe or dangerous to the communities in which a vaccination program being carried out? In a community in which no dengue epidemic exist, for example, no dengue virus of a particular serotype exist, Does the small possibility of introducing that virus into the community where there is no chance of the virus entering into the community through natural means worth the risk? Does the chance of the more virulent form of the dengue fever (dengue hemorrhagic fever DHF or dengue shock syndrome, DSS) occurring in community in which only one serotype of DV is circulating worth the risk? In Thailand, three of the serotypes are circulating, so the use of the vaccine does not impose additional risk. These questions cannot be answered on the basis of the results of our model. We have shown that the use of the vaccine is beneficial.

## Data Availability

The data used to support the findings of this study are available from the corresponding author upon request.

## References

[1] World Health Organization (2017). *Dengue and Severe Dengue: Fact Sheet*.

[2] World Health Organization (2012). *Global Strategy for Dengue Prevention and Control 2012–2020*.

[3] Bureau of Epidemiology, Ministry of Public Health (2016). Annual epidemiology surveillance report 2016.

[4] Esteva L., Vargas C. (1998). Analysis of a dengue disease transmission model. *Mathematical Bioscience*.

[5] Chanprasopchai P., Pongsumpun P., Tang I. M. (2017). Effect of rainfall for the dynamical transmission model of the dengue disease in Thailand. *Computational and Mathematical Methods in Medicine*.

[6] Chanprasopchai P., Pongsumpun P. The transmission dynamic of SIR modeling for dengue fever with vector infection.

[7] Pongsumpun P., Tang I. M. (2003). Transmission of dengue hemorrhagic fever in an age structured population. *Mathematical and Computer Modelling*.

[8] Shim E. (2016). Dengue dynamics and vaccine cost-effectiveness analysis in the Philippines. *American Journal of Tropical Medicine and Hygiene*.

[9] Recker M., Vannice K., Hombach J., Jit M., Simmons C. P. (2016). Assessing dengue vaccination impact: model challenges and future directions. *Vaccine*.

[10] Aguiar M., Stollenwerk N., Halstead S. B. (2016). The impact of the newly licensed dengue vaccine in endemic countries. *PLoS Neglected Tropical Diseases*.

[11] Sridhar S., Luedtke A., Langevin E. (2018). Effect of dengue serostatus on dengue vaccine safety and efficacy. *New England Journal of Medicine*.

[12] Sriprom M., Barbazn P., Tang I. M. (2007). Destabilizing effect of the host immune status on the sequential transmission dynamic of the dengue virus infection. *Mathematical and Computer Modelling*.

[13] Samitivej Hospitals (2016). The first dengue vaccine in Thailand is available at Samitivej hospitals. http://thailand.ahk.de/en/members/member-broadcast/member-broadcast-detail/artikel/the-first-dengue-vaccine-in-thailand-available-at-samitivej-hospitals/?cHash=28e25c2bf27bd4053159b62113ee1733.

[14] Promprou S., Jaroensutasinee M., Jaroensutasinee K. (2005). Climatic factors affecting dengue hemorrhagic fever incidence in Southern Thailand. *Dengue Bulletin*.

[15] Wongkoon S., Jaroensutasinee M., Jaroensutasinee K. (2011). Climatic variability and dengue virus transmission in Chiang Rai, Thailand. *Biomedical*.

[16] Wongkoon S., Jaroensutasinee M., Jaroensutasinee K. (2013). Distribution, seasonal variation & dengue transmission prediction in Sisaket, Thailand. *Indian Journal of Medical Research*.

[17] Wiwanitkit V. (2005). Strong correlation between rainfall and the prevalence of dengue in central region of Thailand in 2004. *Journal of Rural and Tropical Public Health*.

[18] Polwiang S. (2015). The seasonal reproduction number of dengue fever: impacts of climate on transmission. *PeerJ*.

[19] Hopp M. J., Foley J. A. (2003). Worldwide fluctuations in dengue fever cases related to climate variability. *Climate Research*.

[20] Toan T. T., Martens P., Luu N. H., Wright P., Choisy M. (2014). Climatic-driven seasonality of emerging dengue fever in Hanoi, Vietnam. *BMC Public Health*.

[21] Rodrigues H. S., Teresa M., Monteiro T. (2014). Seasonality effects on dengue: basic reproduction number, sensitivity analysis and optimal control. *Mathematical Methods in the Applied Sciences*.

[22] Altizer S., Dobson A., Hosseini P., Hudson P., Pascual M., Rohani P. (2006). Seasonality and the dynamics of infectious diseases. *Ecology Letters*.

